# Supplementary material for: Rendering protein mutation movies with MutAmore
Source: BMC Bioinformatics. 2023 Dec 12;24:469. doi: 10.1186/s12859-023-05610-8 (PMC10714560; doi:10.1186/s12859-023-05610-8)
Supplement: Supplementary file 1 — Additional file 1. In figures S1-S12 we show screenshots of all protein mutation movies used for benchmarking. Corresponding tables S1-S12 indicate runtimes for all individual samples, including prediction time using ESMFold and all MutAmore rendering pipeline steps. Figure S13 visualizes the scaling of runtime for prediction and rendering steps with sequence length. [file 12859_2023_5610_MOESM1_ESM.pdf]

Supporting Online Material (SOM) for:

Rendering protein mutation movies with MutAmore

Konstantin Weissenow[1,2] & Burkhard Rost [1,3]

1 TUM (Technical University of Munich) Department of Informatics, Bioinformatics & Computational Biology - i12, Boltzmannstr. 3, 85748 Garching/Munich, Germany
2 TUM Graduate School, Center of Doctoral Studies in Informatics and its Applications (CeDoSIA), Boltzmannstr. 11, 85748 Garching, Germany
3 Institute for Advanced Study (TUM-IAS), Lichtenbergstr. 2a, 85748 Garching/Munich, Germany & TUM School of Life Sciences Weihenstephan (WZW), Alte Akademie 8, Freising, Germany

Table of Contents for SOM

TABLE OF CONTENTS FOR SOM 1
SHORT DESCRIPTION OF SOM 2
MATERIAL 3
Fig. S1: Protein mutation movie screenshot: Calmodulin-1: 3
Table S1: Runtimes for Calmodulin-1 3
Fig. S2: Protein mutation movie screenshot: Translation initiation factor IF-1: 4
Table S2: Runtimes for Translation initiation factor IF-1 4
Fig. S3: Protein mutation movie screenshot: Translation initiation factor Hras: 5
Table S3: Runtimes for Hras 5
Fig. S4: Protein mutation movie screenshot: Yeast ubiquitin: 6
Table S4: Runtimes for yeast ubiquitin 6
Fig. S5: Protein mutation movie screenshot: Small ubiquitin-related modifier 1: 7
Table S5: Runtimes for small ubiquitin-related modifier 1 7
Fig. S6: Protein mutation movie screenshot: TIM barrel: 8
Table S6: Runtimes for TIM barrel 8
Fig. S7: Protein mutation movie screenshot: Thiamin pyrophosphokinase 1: 9
Table S7: Runtimes for Thiamin pyrophosphokinase 1 9
Fig. S8: Protein mutation movie screenshot: Thiopurine S-methyltransferase: 10
Table S8: Runtimes for Thiopurine S-methyltransferase 10
Fig. S9: Protein mutation movie screenshot: SUMO-conjugating enzyme UBC9: 11
Table S9: Runtimes for SUMO-conjugating enzyme UBC9 11
Fig. S10: Protein mutation movie screenshot: Xylose transporter XylE from E.coli: 12
Table S10: Runtimes for xylose transporter XylE from E.coli 12
Fig. S11: Protein mutation movie screenshot: E.coli autotransporter beta-domain EspP: 13
Table S11: Runtimes for E.coli autotransporter beta-domain EspP 13
Fig. S12: Protein mutation movie screenshot: Colicin I receptor Cir from E.coli: 14
Table S12: Runtimes for Colicin I receptor Cir from E.coli 14
Fig. S13: Prediction runtime increases drastically for longer proteins 15
REFERENCES 15

Short description of SOM

In figures S1-S12 we show screenshots of all protein mutation movies used for benchmarking. Corresponding tables S1-S12 indicate runtimes for all individual samples, including prediction time using ESMFold (Lin et al., 2022) and all *MutAmore* rendering pipeline steps.

Figure S13 visualizes the scaling of runtime for prediction and rendering steps with sequence length.

Material

Fig. S1: Protein mutation movie screenshot: Calmodulin-1:

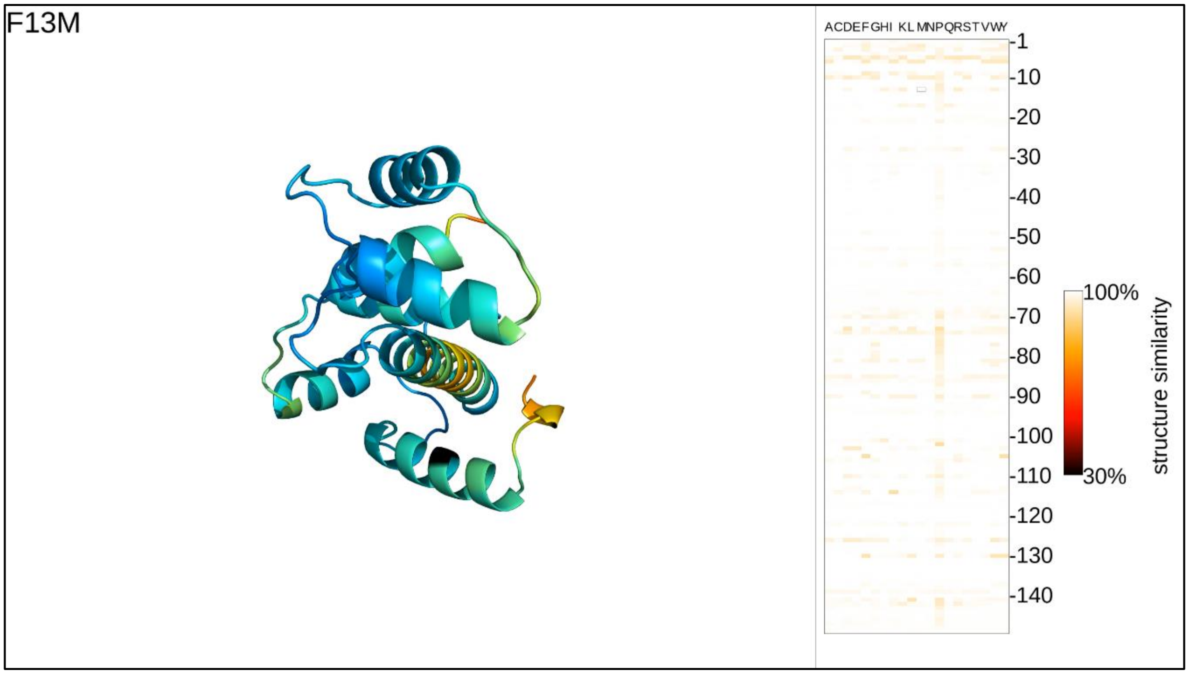

Fig. S1: Protein mutation movie screenshot: Calmodulin-1. Single frame of a protein mutation movie for Calmodulin-1.

Table S1: Runtimes for Calmodulin-1

	Runtime (in sec)	
	720p resolution	4K resolution
Prediction (ESMFold)	5,346	
3D rendering	957	3,389
Structural similarity	116	116
Mutation profile rendering	187	483
Compositing final frames	217	1,350
Movie rendering	9	51
Total (rendering)	1,486	5,389
Total (all)	6,832	10,735

Fig. S2: Protein mutation movie screenshot: Translation initiation factor IF-1:

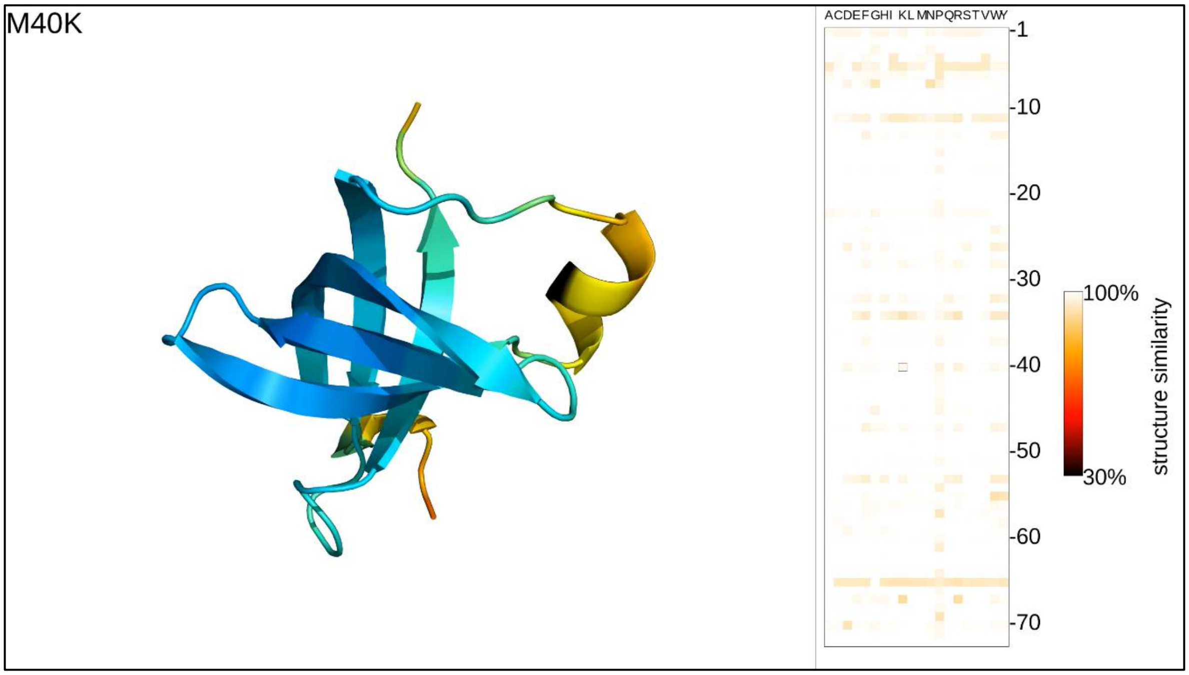

Fig. S2: Protein mutation movie screenshot: Translation initiation factor IF-1. Single frame of a protein mutation movie for Translation initiation factor IF-1.

Table S2: Runtimes for Translation initiation factor IF-1

	Runtime (in sec)	
	720p resolution	4K resolution
Prediction (ESMFold)	591	
3D rendering	357	1,387
Structural similarity	33	34
Mutation profile rendering	63	177
Compositing final frames	97	598
Movie rendering	5	26
Total (rendering)	555	2,222
Total (all)	1,146	2,813

Fig. S3: Protein mutation movie screenshot: Translation initiation factor Hras:

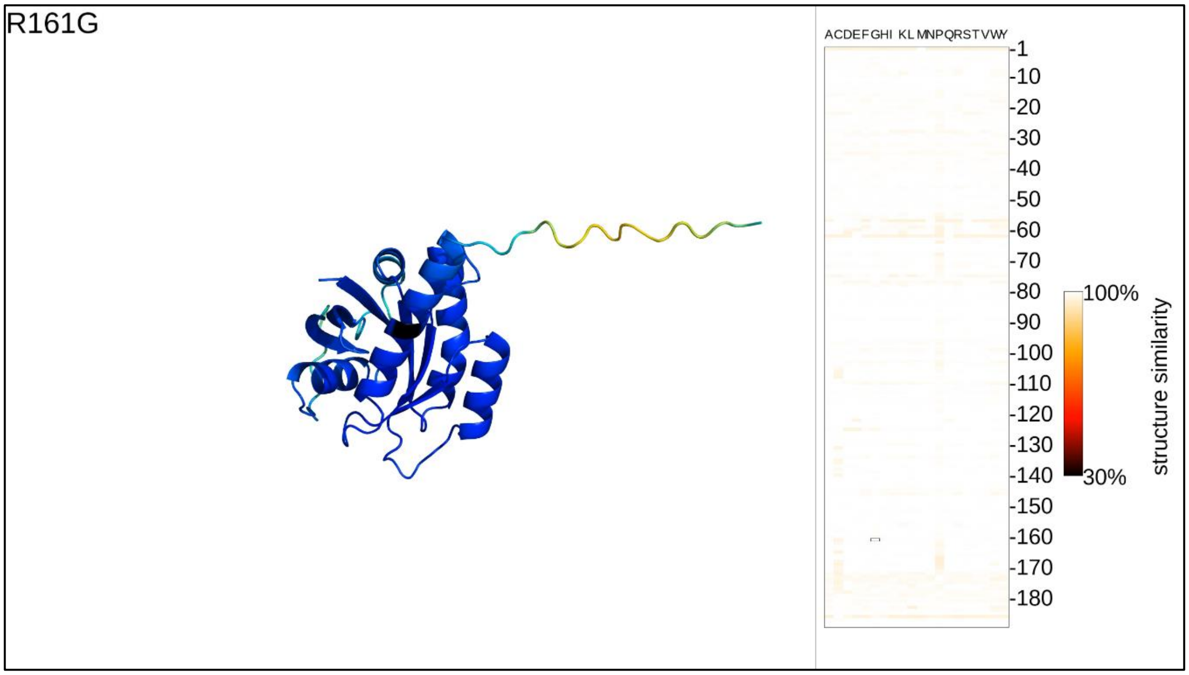

Fig. S3: Protein mutation movie screenshot: Hras. Single frame of a protein mutation movie for Hras.

Table S3: Runtimes for Hras

	Runtime (in sec)	
	720p resolution	4K resolution
Prediction (ESMFold)	11,198	
3D rendering	1,034	3,558
Structural similarity	179	177
Mutation profile rendering	272	704
Compositing final frames	250	1,581
Movie rendering	10	65
Total (rendering)	1,745	6,085
Total (all)	12,943	17,283

Fig. S4: Protein mutation movie screenshot: Yeast ubiquitin:

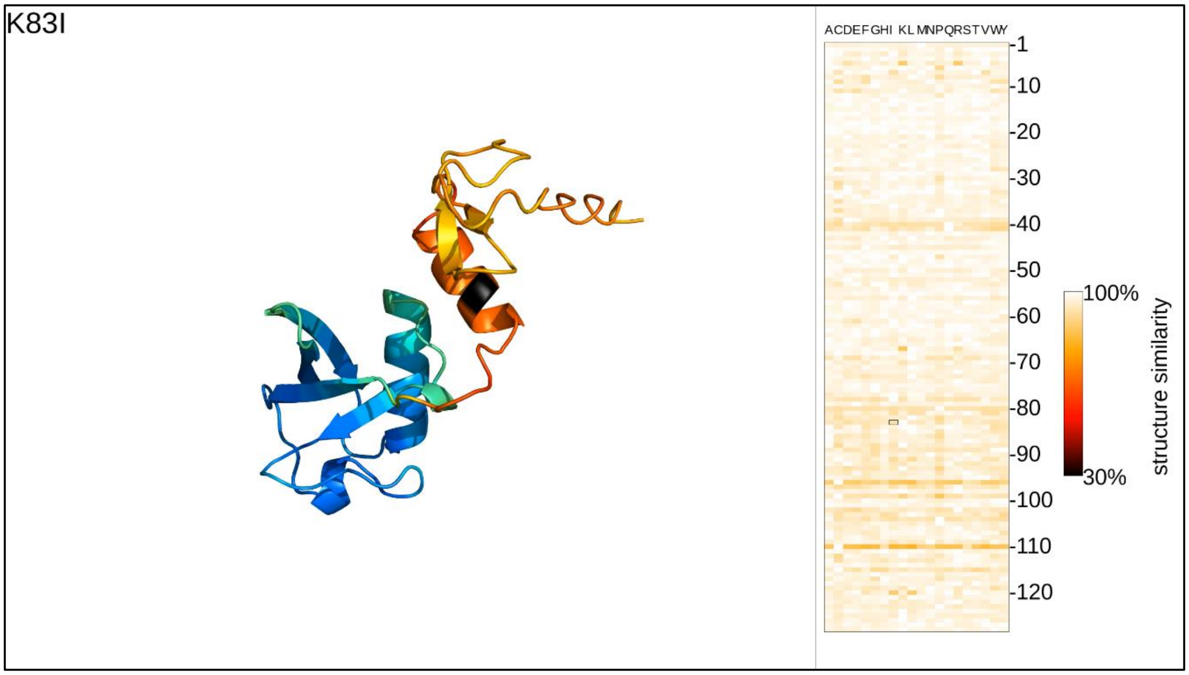

Fig. S4: Protein mutation movie screenshot: Yeast ubiquitin. Single frame of a protein mutation movie for yeast ubiquitin.

Table S4: Runtimes for yeast ubiquitin

	Runtime (in sec)	
	720p resolution	4K resolution
Prediction (ESMFold)	3,226	
3D rendering	709	2,517
Structural similarity	72	88
Mutation profile rendering	147	391
Compositing final frames	153	1,039
Movie rendering	8	49
Total (rendering)	1,089	4,084
Total (all)	4,315	7,310

Fig. S5: Protein mutation movie screenshot: Small ubiquitin-related modifier 1:

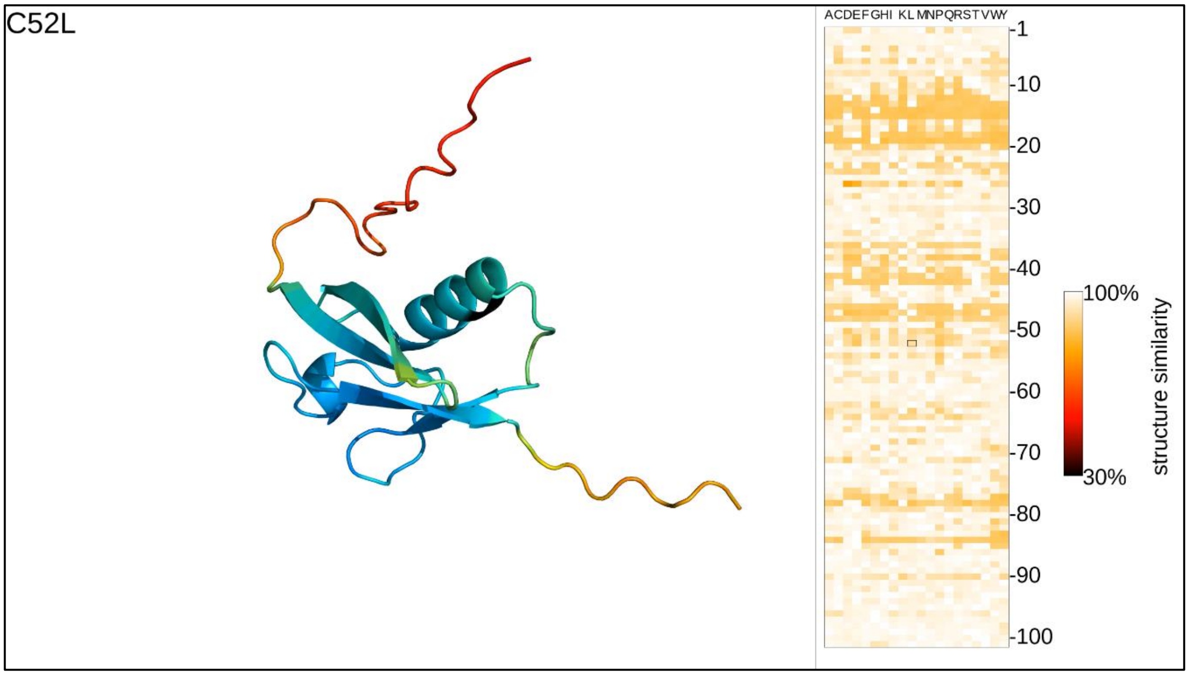

Fig. S5: Protein mutation movie screenshot: Small ubiquitin-related modifier 1. Single frame of a protein mutation movie for Small ubiquitin-related modifier 1.

Table S5: Runtimes for small ubiquitin-related modifier 1

	Runtime (in sec)	
	720p resolution	4K resolution
Prediction (ESMFold)	1,597	
3D rendering	484	1,834
Structural similarity	60	59
Mutation profile rendering	99	288
Compositing final frames	122	815
Movie rendering	6	37
Total (rendering)	771	3,033
Total (all)	2,368	4,630

Fig. S6: Protein mutation movie screenshot: TIM barrel:

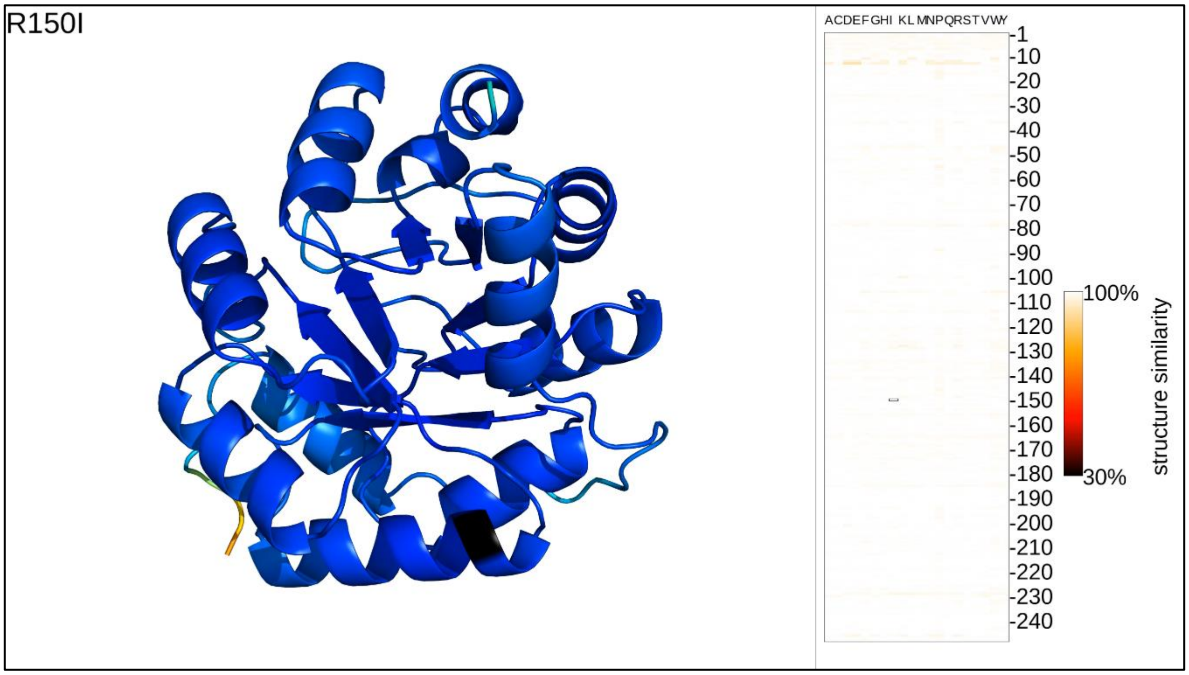

Fig. S6: Protein mutation movie screenshot: TIM barrel. Single frame of a protein mutation movie for TIM barrel.

Table S6: Runtimes for TIM barrel

	Runtime (in sec)	
	720p resolution	4K resolution
Prediction (ESMFold)	28,261	
3D rendering	2,285	9,048
Structural similarity	276	273
Mutation profile rendering	396	1,093
Compositing final frames	464	2,995
Movie rendering	15	86
Total (rendering)	3,436	13,495
Total (all)	31,697	41,756

Fig. S7: Protein mutation movie screenshot: Thiamin pyrophosphokinase 1:

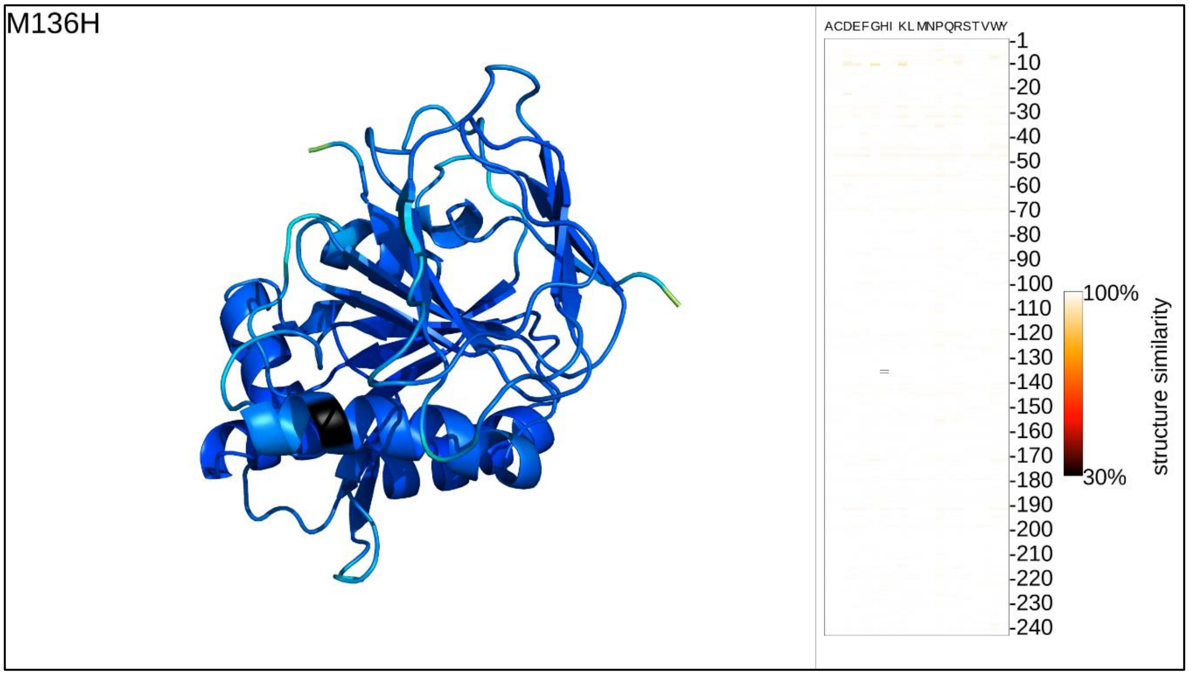

Fig. S7: Protein mutation movie screenshot: Thiamin pyrophosphokinase 1. Single frame of a protein mutation movie for Thiamin pyrophosphokinase 1.

Table S7: Runtimes for Thiamin pyrophosphokinase 1

	Runtime (in sec)	
	720p resolution	4K resolution
Prediction (ESMFold)	26,481	
3D rendering	2,042	6,969
Structural similarity	271	273
Mutation profile rendering	398	1,075
Compositing final frames	387	2,603
Movie rendering	14	80
Total (rendering)	3,112	11,000
Total (all)	29,593	37,481

Fig. S8: Protein mutation movie screenshot: Thiopurine S-methyltransferase:

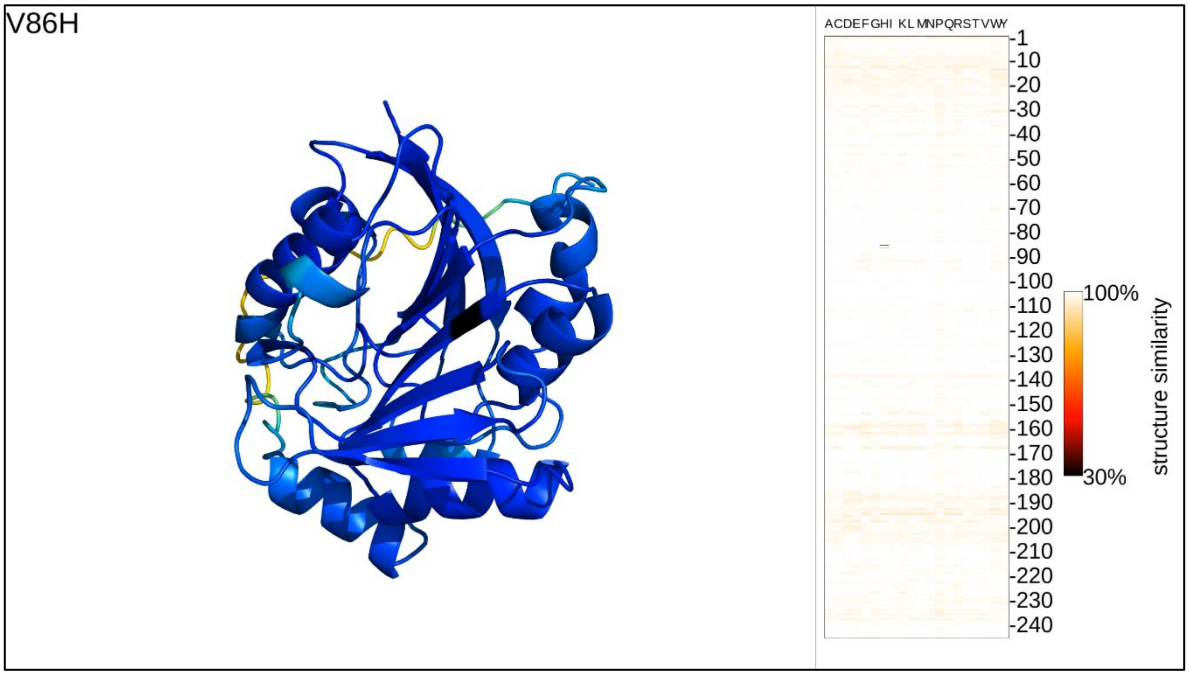

Fig. S8: Protein mutation movie screenshot: Thiopurine S-methyltransferase. Single frame of a protein mutation movie for Thiopurine S-methyltransferase.

Table S8: Runtimes for Thiopurine S-methyltransferase

	Runtime (in sec)	
	720p resolution	4K resolution
Prediction (ESMFold)	27,296	
3D rendering	1,986	6,656
Structural similarity	284	286
Mutation profile rendering	402	1,086
Compositing final frames	384	2,494
Movie rendering	15	83
Total (rendering)	3,071	10,605
Total (all)	30,367	37,901

Fig. S9: Protein mutation movie screenshot: SUMO-conjugating enzyme UBC9:

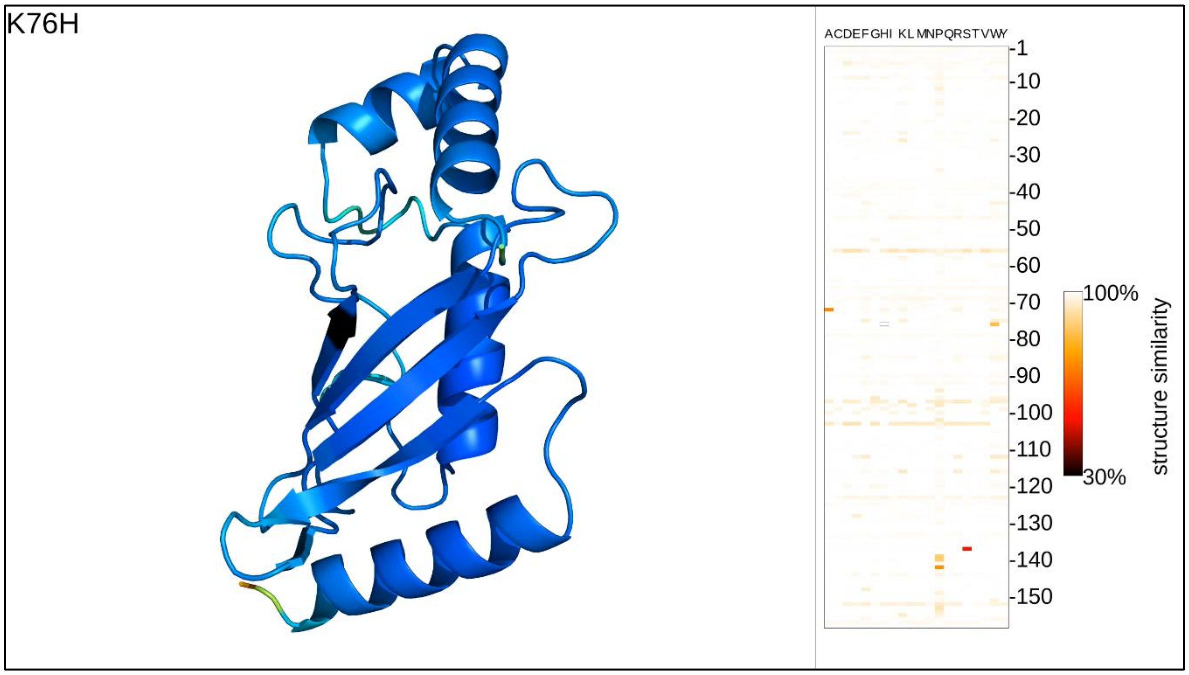

Fig. S9: Protein mutation movie screenshot: SUMO-conjugating enzyme UBC9. Single frame of a protein mutation movie for SUMO-conjugating enzyme UBC9.

Table S9: Runtimes for SUMO-conjugating enzyme UBC9

	Runtime (in sec)	
	720p resolution	4K resolution
Prediction (ESMFold)	6,338	
3D rendering	1,071	4,018
Structural similarity	121	134
Mutation profile rendering	197	533
Compositing final frames	235	1,675
Movie rendering	10	55
Total (rendering)	1,634	6,415
Total (all)	7,972	12,753

Fig. S10: Protein mutation movie screenshot: Xylose transporter XylE from E.coli:

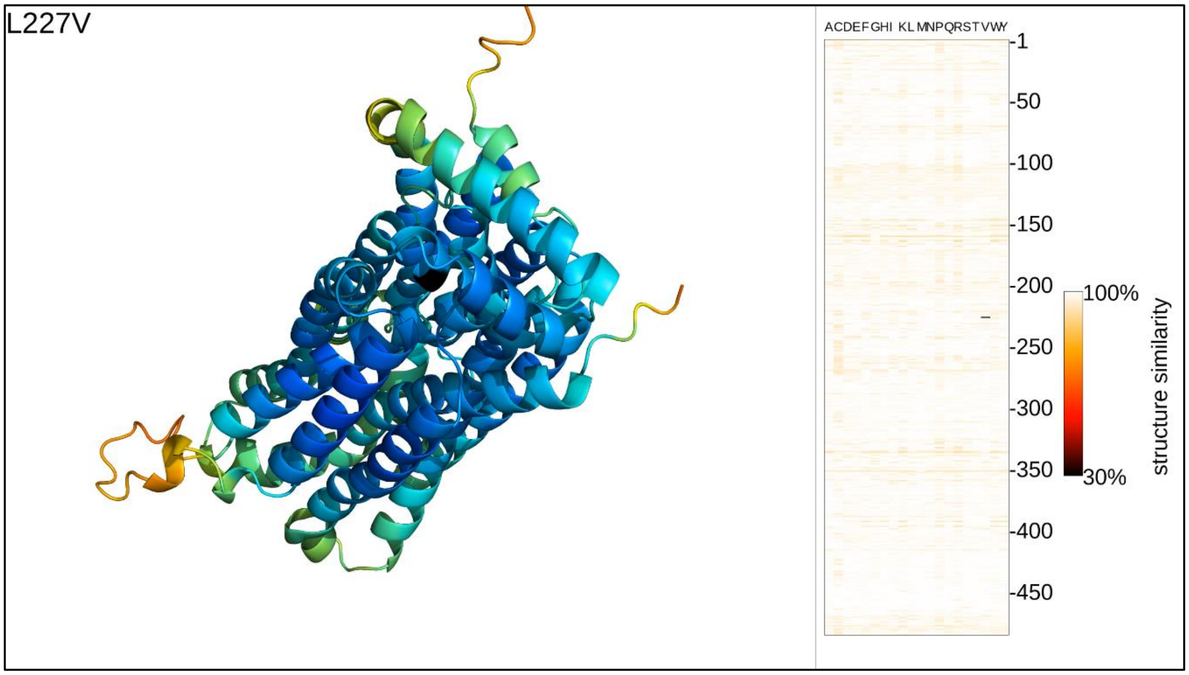

Fig. S10: Protein mutation movie screenshot: Xylose transporter XylE from E.coli. Single frame of a protein mutation movie for the xylose transporter XylE from E.coli.

Table S10: Runtimes for xylose transporter XylE from E.coli

	Runtime (in sec)	
	720p resolution	4K resolution
Prediction (ESMFold)	321,716	
3D rendering	5,895	25,471
Structural similarity	839	976
Mutation profile rendering	683	4,267
Compositing final frames	853	7,208
Movie rendering	30	168
Total (rendering)	8,300	38,090
Total (all)	330,016	359,806

Fig. S11: Protein mutation movie screenshot: E.coli autotransporter beta-domain EspP:

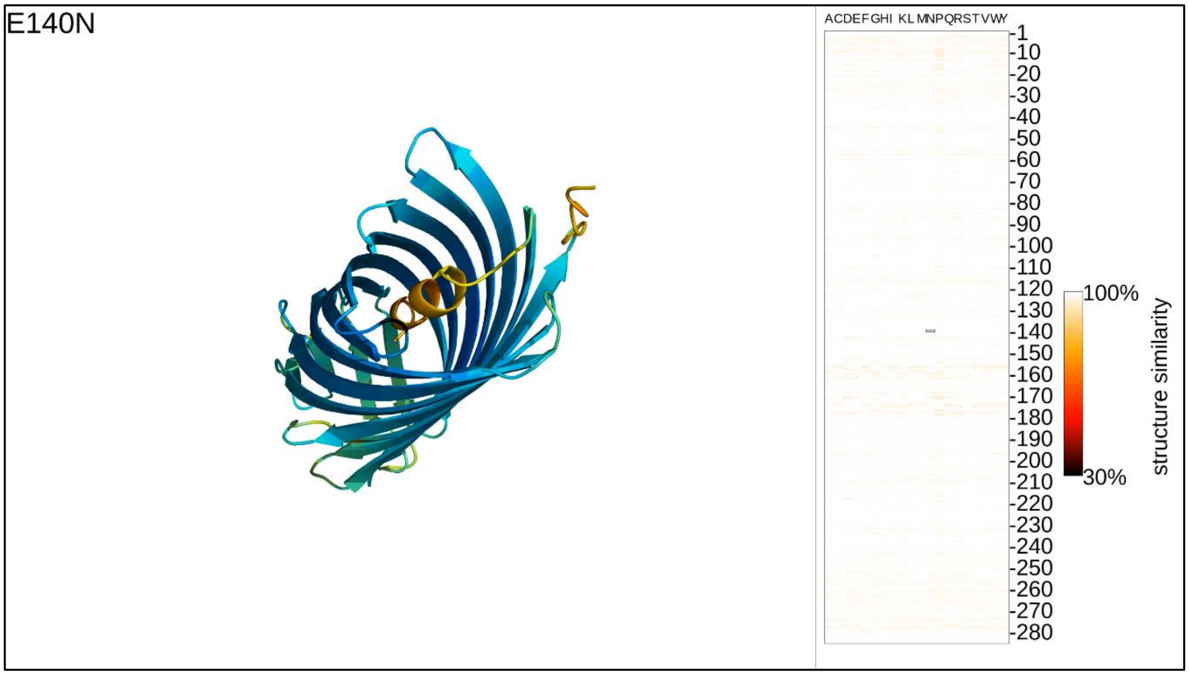

Fig. S11: Protein mutation movie screenshot: E.coli autotransporter beta-domain EspP. Single frame of a protein mutation movie for E.coli autotransporter beta-domain EspP.

Table S11: Runtimes for E.coli autotransporter beta-domain EspP

	Runtime (in sec)	
	720p resolution	4K resolution
Prediction (ESMFold)	47,429	
3D rendering	2,070	8,659
Structural similarity	345	386
Mutation profile rendering	499	1,557
Compositing final frames	385	2,943
Movie rendering	15	110
Total (rendering)	3,314	13,655
Total (all)	50,743	61,084

Fig. S12: Protein mutation movie screenshot: Colicin I receptor Cir from E.coli:

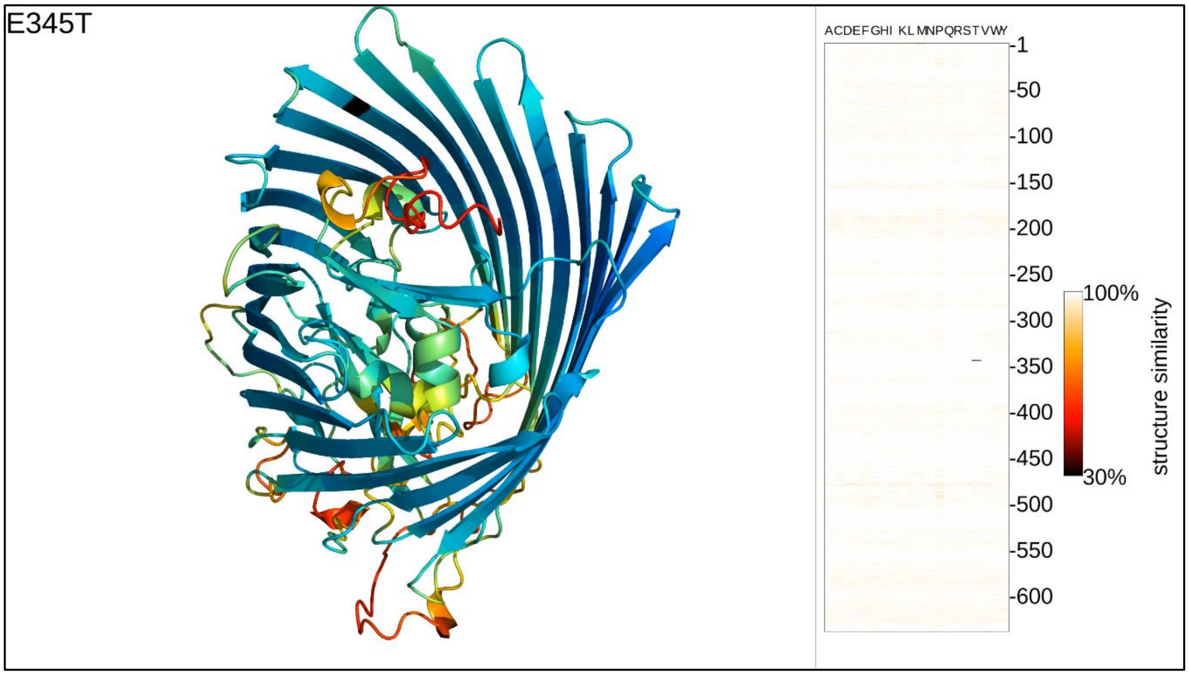

Fig. S12: Protein mutation movie screenshot: Colicin I receptor Cir from E.coli. Single frame of a protein mutation movie for Colicin I receptor Cir from E.coli.

Table S12: Runtimes for Colicin I receptor Cir from E.coli

	Runtime (in sec)	
	720p resolution	4K resolution
Prediction (ESMFold)	944,335	
3D rendering	8,003	33,323
Structural similarity	1,412	1,422
Mutation profile rendering	1,129	5,217
Compositing final frames	1,183	7,731
Movie rendering	35	202
Total (rendering)	11,762	47,895
Total (all)	956,097	992,230

Fig. S13: Prediction runtime increases drastically for longer proteins

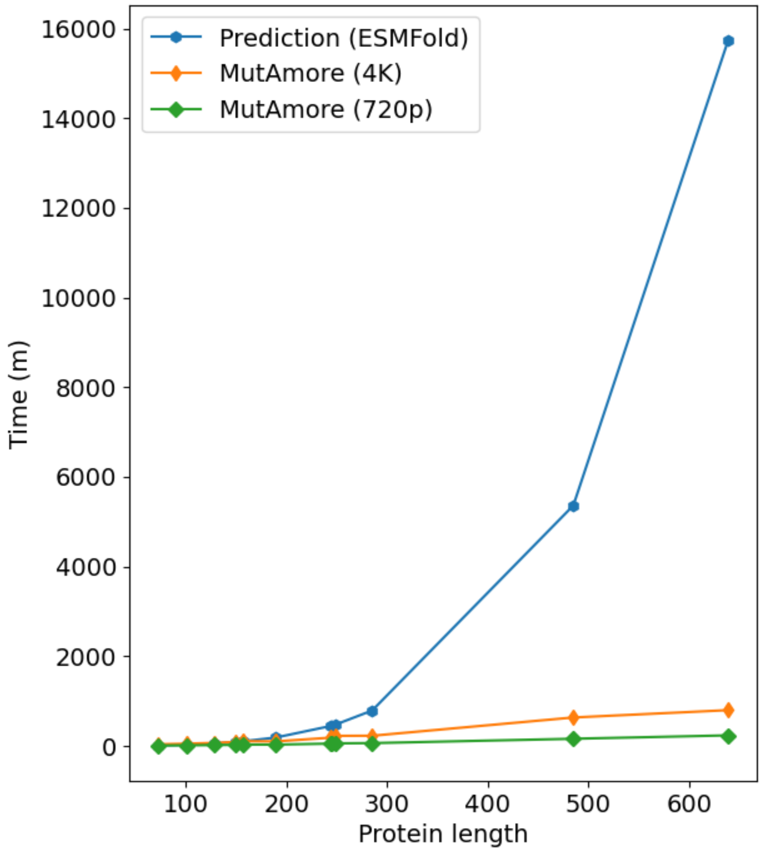

Fig. S13: Prediction runtime increases drastically for longer proteins. Mutations for shorter proteins (x-axis; length in residues) can be predicted at high speeds by ESMFold (y-axis; runtime in seconds). For the shortest proteins in the benchmark, MutAmore rendering steps in high resolution (4K) take slightly longer than computing structure predictions. However, above a length of about 200 residues, ESMFold's runtime increases considerably, due to (a) the inability to batch multiple mutant sequences in one computation pass and (b) the general increase in computational complexity and memory consumption of its architecture for longer sequences.

References

Lin, Z., Akin, H., Rao, R., Hie, B., Zhu, Z., Lu, W., Santos Costa, A. d., Fazel-Zarandi, M., Sercu, T., Candido, S., & Rives, A. (2022). Language models of protein sequences at the scale of evolution enable accurate structure prediction. *bioRxiv*, 2022.2007.2020.500902. https://doi.org/10.1101/2022.07.20.500902
